# A Multivariate Approach to Study the Bacterial Diversity Associated to the Wooden Shelves Used for Aging Traditional Sicilian Cheeses

**DOI:** 10.3390/foods11050774

**Published:** 2022-03-07

**Authors:** Raimondo Gaglio, Gabriele Busetta, Riccardo Gannuscio, Luca Settanni, Giuseppe Licitra, Massimo Todaro

**Affiliations:** 1 Dipartimento Scienze Agrarie, Alimentari e Forestali, Università degli Studi di Palermo, Viale delle Scienze, Ed. 5, 90128 Palermo, Italy; raimondo.gaglio@unipa.it (R.G.); gabriele.busetta@unipa.it (G.B.); riccardo.gannuscio@unipa.it (R.G.); massimo.todaro@unipa.it (M.T.); 2 Dipartimento di Agricoltura, Alimentazione e Ambiente (Di3A), Università degli Studi di Catania, Via Valdisavoia 5, 95123 Catania, Italy; glicitra@unict.it

**Keywords:** cheese microbiology, cheese ripening, lactic acid bacteria, MiSeq Illumina, statistical analysis, traditional cheeses, wooden shelves

## Abstract

The present study was carried to correlate the microbial diversity of the biofilms developed on the wooden boards used for aging traditional Sicilian cheeses with cheese typology. To this end, the microbial diversity of the shelves in contact with the cheeses PDO Pecorino Siciliano, PDO Piacentinu Ennese, and TAP Caciocavallo Palermitano, during ripening, was evaluated by a multivariate statistical approach. The shelf biofilms of this study were previously analyzed for their microbial composition, but no correlation between biodiversity and cheese type was investigated. Canonical discriminant analysis confirmed a cheese typology effect on the microbial loads of the wooden shelves investigated. Regarding the plate count data, the centroids of different cheeses were statistically distant from one another. This analysis also showed a good graphic separation of data regarding bacterial order operational taxonomy units (OTUs). Thus, the microbiological differences imputed to the cheese typologies were not affected by the environmental conditions of the facilities. Furthermore, wooden shelf lactic acid bacteria (LAB) were investigated for their ability to inhibit the main dairy pathogens. Although inhibitors were mainly enterococci, *P. pentosaceus* WS287 and *W. paramesenteroides* WS581 showed the highest inhibition activity, indicating their possible application to control the undesired bacteria in situ.

## 1. Introduction

Wood has represented the main material for the manufacture of dairy equipment in Europe for centuries [1]. In Sicily (southern Italy), the cheese making process remained almost unvaried over time for several cheese productions [2], most of them enjoy quality status such as protected denomination of origin (PDO) or other recognitions by the European Union [3,4]. Although there is no specific contraindication regarding the use of wood to process foods as in European Regulation (EC) no. 1935/2004 [5], the direct contact of food matrices with wood is still controversial in the majority of EU Countries. In Italy, dairy production can be carried out with wooden equipment thanks to regulation EC no. 2074/2005, which derogates from the EC no. 852/2004 for foods with traditional characteristics [6].

The controversial aspects of the use of wood in food production is due to its porous structure, which facilitate bacterial trapping. Once bacteria are adsorbed onto the wood surface, they might develop biofilms, becoming resident communities and, consequently, contaminating food products [7]. In the case of dairy products, specifically cheeses, several works have demonstrated how microbial biofilms formed on the surface of wooden vats used for milk processing are responsible for curd acidification, cheese ripening, and even the inhibition of undesired bacteria attachment and development thanks to the massive presence of lactic acid bacteria (LAB) [8,9,10,11,12,13,14]. Recently, another wood equipment, the open-topped table (namely “mastredda”) used for curd acidification during Sicilian stretched cheese production, was analyzed for the composition of LAB biofilms, evidencing the presence of *Enterococcus*, *Lactobacillus*, *Lacticaseibacillus*, *Lactiplantibacillus*, *Levilactobacillus*, *Lactococcus*, *Leuconostoc*, *Pediococcus*, and *Streptococcus* [15].

Nowadays, there is renewed interest towards the safety aspects of wood in contact with cheese during ripening. In fact, the US Food and Drug Administration (FDA) expressed some concerns about cheeses ripened on wooden planks for the possible transfer of pathogenic bacteria, such as *Listeria monocytogenes* [16]. This aspect is often highlighted by detractors of traditional productions, without an in-depth consideration of the barrier effect of the resident microorganisms of a given wood biofilm against foodborne pathogens. Although limited research is available on this topic, some evidence has been provided. Mariani et al. [17,18] characterized the microbial populations of the wooden shelves used to ripen French smear cheese, PDO Reblochon de Savoie, reporting the massive presence of micrococci-corynebacteria, yeasts, and molds, as well as LAB, *Staphylococcus*, and *Pseudomonas*, and evaluated the bio-protective action of the wooden shelf biofilms, specifically against *L. monocytogenes*. Regarding the microbial characterization, Guzzon et al. [19] studied the biofilms of the wooden shelves used for the ripening of another smear cheese, the Italian PDO Fontina, and evidenced a cause–effect relationships between the dominant Actinobacteria populations and a red-brown pigmentation defect. The microbiota of wooden boards used for cheese ripening has been also recently considered in the US by Wadhawan et al. [20], who detected Actinobacteria, Firmicutes, and Proteobacteria at dominant levels, and highlighted the consistent presence of halophilic bacteria. The massive presence of halophilic and moderately halophilic bacteria was also revealed among biofilms of the wooden shelves used for aging traditional Sicilian cheeses, while the presence of the main dairy pathogens, such as coagulase-positive staphylococci (CSP), *Salmonella* spp., and *L. monocytogenes*, was not found [21].

Considering that only a few studies have been carried out on the microbial diversity of the wooden boards used for cheese aging, the main limitations in this field are the low amount of data regarding cell densities and the taxon composition/distribution of the biofilms among traditional cheese productions, and the complete lack of information about the inhibitory properties of these biofilms towards *Salmonella* spp., *Escherichia coli*, and CPS.

This study is part of a project aimed to characterize the production processes of natural and historic cheeses of Southern Italy, specifically PDO Pecorino Siciliano, PDO Piacentinu Ennese, and traditional agri-food product (TAP) Caciocavallo Palermitano. PDO Pecorino Siciliano cheese is a hard cheese produced with raw milk of autochthonous Sicilian sheep breeds Valle del Belìce, Comisana, and Pinzirita, reared under an extensive system [22]. PDO Piacentinu Ennese cheese is a semi-hard product of a restricted area (Enna province) in central Sicily. In addition, this cheese is made from raw milk of the sheep breeds Valle del Belìce, Comisana, and Pinzirita, reared under an extensive system, but unlike PDO Pecorino Siciliano cheese, Sicilian saffron and black pepper are added during the manufacture [23]. TAP Caciocavallo Palermitano is a hard cheese produced within the Palermo province. It is a stretched cheese obtained from raw bovine milk of Cinisara, Pezzata Rossa, and Bruna breeds and half-breeds [24].

The objectives of the present research were to analyze the results of culture-dependent and to perform independent microbiological investigations of the wooden shelves used for ripening the three medium-aged cheeses reported above, using a multivariate statistical approach to find relationships between wooden shelf bacterial diversity and cheese typology, as well as to characterize the inhibitory potential of wooden board LAB against *L. monocytogenes*, *Salmonella* spp., *Escherichia coli*, and CPS.

## 2. Materials and Methods

### 2.1. Collection of Wooden Shelf Biofilms and Microbiological Investigation

The microbial biofilms associated to the wooden shelves used to ripen PDO Pecorino Siciliano cheese, PDO Piacentinu Ennese cheese and TAP Caciocavallo Palermitano cheese were collected within the provinces of Agrigento, Enna, Palermo and Trapani (central and western Sicily, Italy). During cheese ripening, 18 shelves were sampled from 18 dairy facilities. The contact of the boards with the cheese rind lasted 1–2 months for the PDO Piacentinu Ennese cheese, and 4–5 months for the other two cheeses. A square surface (100 cm^2^) of each board was subjected to a nondestructive microbial collection through brushing. Shelf biofilms were finally collected with a sterile gauze and were transferred into a Durham bottle containing 100 mL of Ringer’s solution (Sigma-Aldrich, Milan, Italy). 

All wooden shelves were microbiologically investigated by culture-dependent and -independent methods, as reported by Settanni et al. [21]. Briefly, several microbial groups were detected, as follows: total mesophilic microorganisms (TMM) on plate count agar (PCA) incubated at 30 °C for 72 h; members of the Enterobacteriaceae family on violet red bile glucose agar (VRBGA) incubated at 37 °C for 24 h; total coliforms (TC) on violet red bile agar (VRBA) incubated at 37 °C for 24 h; Escherichia coli on Hektoen enteric agar (HEA) incubated at 37 °C for 24 h; pseudomonads on *Pseudomonas* agar base (PAB) and incubated at 25 °C for 48 h; mesophilic and thermophilic LAB cocci on Medium 17 (M17) agar incubated at 30 and 44 °C, respectively, for 48 h; mesophilic and thermophilic LAB rods on de Man–Rogosa–Sharpe (MRS) agar incubated at 30 and 44 °C, respectively, for 48 h; enterococci on Kanamycin Esculin Azide (KEA) agar incubated at 37 °C for 24 h; yeasts on dichloran Rose Bengal chloramphenicol (DRBC) agar incubated at 28 °C for 48 h; and molds on potato dextrose agar (PDA) incubated at 25 °C for 7 days.

Regarding the culture-independent approach applied, DNAs from biofilms were extracted using the Power Food Microbial DNA isolation kit (Mo Bio Laboratories, Inc., Carlsbad, CA, USA), purified by PowerClean DNA Cleanup kit (Mo Bio Laboratories, Inc.) and quantified through Nanodrop 8800 fluorospectrometer (Thermo Scientific, Wilmington, NC, USA). A 464-nucleotide sequence of the V3-V4 region of the 16S rRNA gene (*E. coli* positions 341 to 805) was amplified from the total DNA of each sample and was paired-end sequenced by the Illumina MiSeq system. Raw paired-end FASTQ files were demultiplexed using idemp (https://github.com/yhwu/idemp/blob/master/idemp.cpp, accessed on 25 March 2021) and imported into Quantitative Insights Into Microbial Ecology (Qiime2, version 2018.2). The sequences were then filtered, trimmed, denoised, and merged using DADA2 (87). The chimeric sequences were removed via the consensus method in DADA2. Taxonomic and compositional analyses were carried on using the plugins feature-classifier (https://github.com/qiime2/q2-feature-classifier, accessed on 25 March 2021). A pretrained naive Bayes classifier based on the Greengenes 13_8 99% Operational Taxonomic Units (OTUs) database was applied to the paired-end sequence reads to generate taxonomy tables.

In the present work, all data were reported per cheese typology.

### 2.2. Antagonistic Activity of LAB

The antibacterial activity of the wooden shelf LAB isolated and identified by Settanni et al. [21] was evaluated against the four main dairy pathogens: *Escherichia coli*, *L. monocytogenes, Salmonella* Enteritidis, and *Staphylococcus aureus*. The indicator (sensitive) bacteria used in this study were provided by the American Type Culture Collection: *E. coli* ATCC25922, *L. monocytogenes* ATCC 19114, *S.* Enteritidis ATCC13076, and *St. aureus* ATCC33862. All of the indicators were reactivated in Brain Heart Infusion (BHI) broth (Oxoid) at 37 °C for 24 h before testing the antagonistic activity of the LAB. The inhibitory assay was conducted as reported by Corsetti et al. [25] using the well diffusion assay. The assays were performed in duplicate.

### 2.3. Statistical Analyses

The microbial loads of the wooden shelf biofilms to logarithmic transformation were subjected in order to normalize the distribution, while for the data of the culture-independent microbiological analysis, which presented a Poisson distribution with a large number of zeros, a square root transformation applied to y + 0.5, was carried out [26]. Box and whisker plots of microbial loads (Log CFU/cm^2^) of TMM, Enterococci, and LAB biofilms developed on the wooden shelves used to ripen traditional Sicilian cheeses were carried out. On the transformed data, a dual statistical approach was applied—univariate and multivariate analyses. An ANOVA model was used to test the effect of the type of cheese (PDO Pecorino Siciliano, PDO Piacentinu Ennese, and TAP Caciocavallo Palermitano) on the dependent variables, while the multivariate analysis was carried out with a Canonical discriminant analysis (GLM and CANDISC procedures of SAS 9.1.2 software, 2004). The degree of dissimilarity among cheeses’ wooden shelves was measured using squared Mahalanobis distances (MD), and the reliability of the canonical discriminant model was finally assessed by cross-validation, where the statistical tests used were Wilks Lambda, Pillai, Hotelling−Lawley, and Roy maximum root.

## 3. Results

### 3.1. Microbial Loads of Wooden Shelf Biofilms

The results of the plate counts of the 12 main microbial groups associated with dairy productions are reported in Table 1. Statistically significant differences among wooden shelves were found for the levels of almost all microbial groups investigated, with the exception of mesophilic and thermophilic LAB cocci.

The PDO Pecorino Siciliano cheese wooden shelves showed the highest loads of TMM, members of Enterobacteriaceae family, total coliforms, and *E. coli*. The wooden boards used for TAP Caciocavallo Palermitano cheese ripening displayed the lowest loads of pseudomonads, while those used for PDO Piacentinu Ennese cheese showed the highest loads of enterococci. Regarding the other LAB groups, significant differences were registered among rod populations. To this purpose, the wooden shelves used for PDO Pecorino Siciliano cheese ripening displayed the highest loads for mesophilic species and the lowest for the thermophilic ones.

Yeasts were present at consistent levels on the wooden boards analysed. In particular, yeast loads of ovine milk cheeses (PDO Pecorino Siciliano and PDO Piacentinu Ennese cheeses) were significantly higher than those of the. TAP Caciocavallo Palermitano cheeses processed from cow’s milk. The most abundant levels of molds were detected for the wooden shelves used for the PDO Pecorino Siciliano cheese, but the differences with those used for the TAP Caciocavallo Palermitano cheeses were not statistically significant.

**Figure 1:**
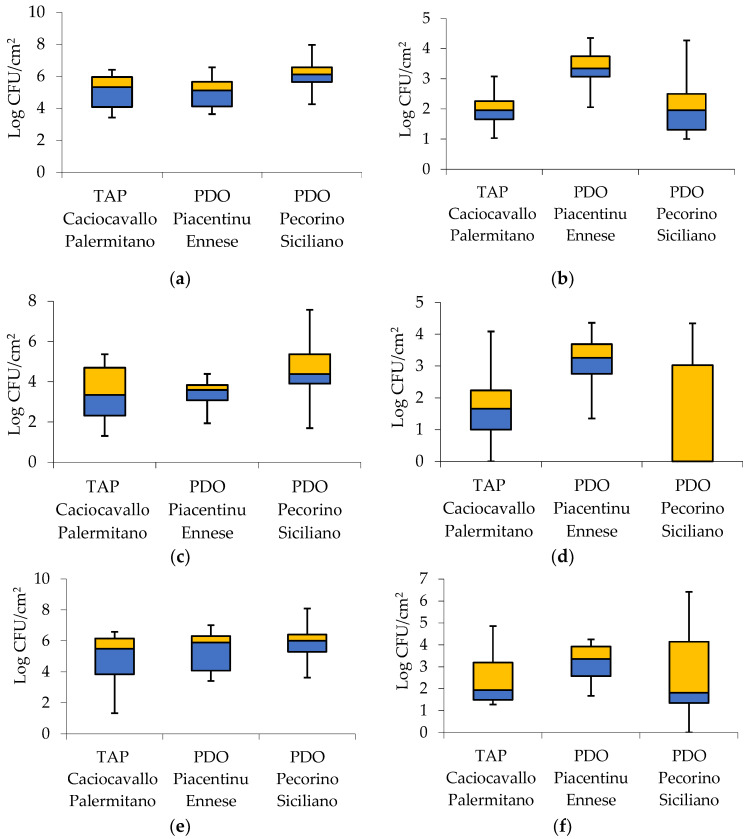
Box and whisker plots of microbial loads of biofilms developed on the wooden shelves used to ripen traditional Sicilian cheeses: (**a**) total mesophilic microorganisms (TMM), (**b**) enterococci, (**c**) mesophilic LAB rods, (**d**) thermophilic LAB rods, (**e**) mesophilic LAB cocci, (**f**) thermophilic LAB cocci.

The distribution of LAB, enterococci, and TMM loads is reported as box and whisker plots in Figure 1.

The TMM variability observed for the PDO Pecorino Siciliano wooden shelves was lower than that registered for the boards used for the other two cheeses (Figure 1a). On the contrary, the TMM of the boards used for the Pecorino Siciliano cheese showed the highest levels. The enterococci distribution of PDO Piacentinu Ennese wooden shelves (Figure 1b) showed the highest levels. The distribution of the levels of mesophilic LAB rods (Figure 1c) showed the highest variability in the PDO Pecorino Siciliano wooden shelves. In the case of thermophilic LAB rods (Figure 1d), the distribution was quite wide for the very low levels (below detection limit) revealed on the wooden shelves used to ripen TAP Caciocavallo Palermitano and PDO Pecorino Siciliano cheeses. The mesophilic LAB cocci distribution (Figure 1e) was highly similar among the wooden shelves analyzed. Regarding termophilic LAB cocci (Figure 1f), the widest distribution was observed for the PDO Pecorino Siciliano wooden shelves.

**Figure 2:**
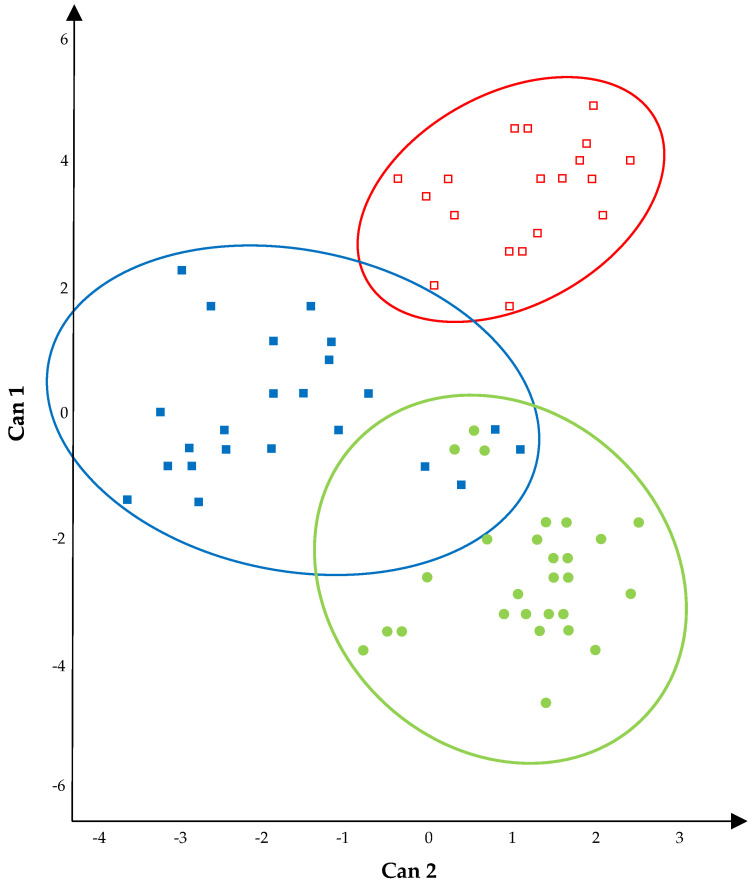
Plot of Canonical 1 × Canonical 2–variable: microbial loads of wooden shelves’ biofilm. ▪, wooden shelves used for the ripening of TAP Caciocavallo Palermitano cheese; ●, wooden shelves used for the ripening of PDO Pecorino Siciliano cheese; ▫, wooden shelves used for the ripening of Piacentinu Ennese cheese. Centroids coordinates: ▪ (0.04; −1.90); ● (−2.5; 0.93); ▫ (3.46; 0.97).

The multivariate approach confirmed the discrimination among the results described above. Statistical tests (Wilks Lambda, Pillai, Hotelling−Lawley, and Roy maximum root) on canonical discriminant analysis (Figure 2) confirmed the effect of cheese typology on the microbial loads of the wooden shelves used for ripening.

The MD between the three centroids of Figure 2 was statistically significant. The longest distance was registered between the wooden shelves of the PDO Pecorino Siciliano cheese area and those of the PDO Piacentinu Ennese cheese (MD = 29.04; *p* < 0.001). On the contrary, the shortest distance was measured between the centroid area of PDO Pecorino Siciliano wooden shelves and that of TAP Caciocavallo Palermitano wooden shelves (MD = 13.10; *p* < 0.001), for which a slight overlap of some points was also observed.

The distances observed among the three centroids were mainly due to the first canonical variable (vertical axis of Figure 1). In particular, thermophilic LAB rods were positively correlated, while mesophilic LAB rods were negatively correlated considering this canonical variable (Table 2).

The LAB rod levels mostly influenced the separation of wooden shelves per cheese typology. This separation was also clear with regards to the second canonical variable, especially concerning the differences between the wooden shelves used for ripening ovine cheeses and those used for TAP Caciocavallo Palermitano cheeses. With the second canonical variable, thermophilic LAB rod counts were negatively correlated, while the loads of the pseudomonads were positively correlated.

### 3.2. Culture-Independent Microbiological Analysis

The taxonomy classification allowed for identifying 14 phyla, 32 classes, 52 orders, 93 families, and 137 genera from the wooden shelf biofilms [21]. In the present study, the operational taxonomy units (OTUs) with a relative abundance >0.1%, were grouped per order (Table 3) and were processed by multivariate statistical analysis. In order to elaborate the relative abundance values, a square root transformation Y=√((x)+0.5) was applied to normalize the data presenting a Poisson distribution.

The bacterial orders present at the highest relative abundances were Actinomycetales (19.04–34.78%) and Bacillales (16.39–43.72%), followed by Lactobacillales (4.17–9.05%). The effect of the cheese typology on the bacterial composition of the wooden shelves was statistically significant for Flavobacteriales (*p* < 0.001) and Bacillales (*p* < 0.05). In particular, the wooden shelves of the PDO Piacentinu Ennese cheese showed a consistently higher presence of Flavobacteriales (6.84%) than those of the TAP Caciocavallo Palermitano cheese (0.11%) and PDO Pecorino Siciliano cheese (0.29%). Regarding the Bacillales order, the TAP Caciocavallo Palermitano cheese wooden shelves displayed the highest relative abundance and the differences found for the other wooden shelves used in the ripening of the other two cheeses (both from ovine milk) were not statistically significant. No statistical differences were found among cheese wooden shelves regarding the other orders identified, probably due to low sample size and/or to a high variability of the relative abundance of the OTUs.

**Figure 3:**
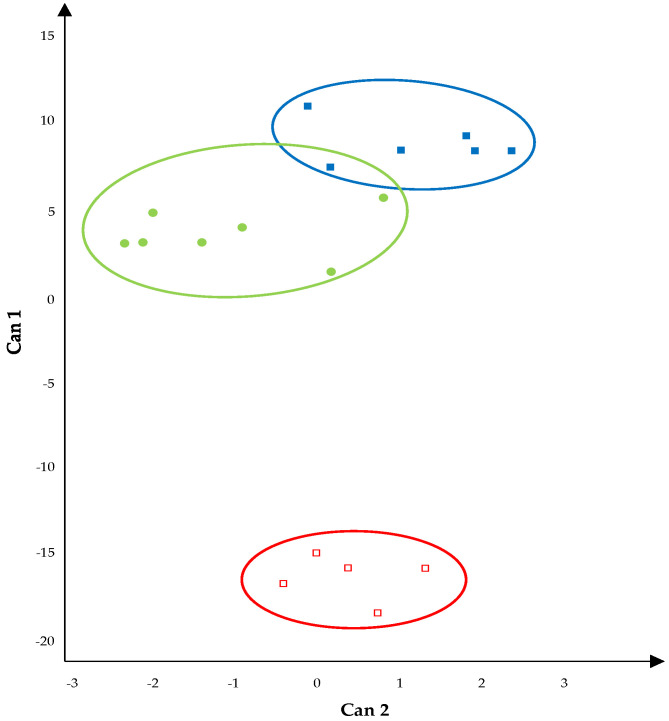
Plot of Canonical 1 × Canonical 2–variable: relative abundance of OTUs detected on wooden shelves biofilm. Symbols: ▪, wooden shelves used for the ripening of TAP Caciocavallo Palermitano cheese; ●, wooden shelves used for the ripening of PDO Pecorino Siciliano cheese; ▫, wooden shelves used for the ripening of Piacentinu Ennese cheese. Centroids coordinates: ▪ (8.61; −1.07); ● (−15.74; 0.31); ▫ (3.85; −1.14).

The OTUs’ relative abundances were subjected to the multivariate statistical analysis. Not all of the results of the statistical tests obtained with the CANDISC procedure were significant: the Wilks Lambda and Hotelling−Lawley tests showed a significance for *p* < 0.05, while the Roy maximum root test was significant for *p* < 0.01. The canonical 1 x canonical 2 plot (Figure 3) showed a good graphic separation between the three areas, with a clear remark between the wooden shelves used to ripen the PDO Piacentinu Ennese cheeses and those used for the ripening of the other two cheeses.

The graphical observation was also confirmed by MD, which was only statistically significant between the PDO Piacentinu Ennese cheese versus Caciocavallo Palermitano PAT cheese wooden shelves (MD = 594; *p* < 0.01), and between the PDO Piacentinu Ennese cheese versus Pecorino Siciliano PDO cheese wooden shelves (MD = 386; *p* < 0.05).

The clear separation between the wooden shelves used for the Piacentinu Ennese cheese ripening and all of the other wooden shelves can be observed on the ordinate axis, which corresponds to first canonical variable, which explain the 99% total variability. With regards to the first canonical variable, the Rhizobiales, Bacillales, Actinomycetales, and Lactobacillales orders were positively correlated, while with Flavobacteriales, Sphingobacteriales, Alteromonadales, and Salinisphaerales the orders were negatively correlated (Table 4).

### 3.3. Inhibitory Activity of LAB

**Figure 4:**
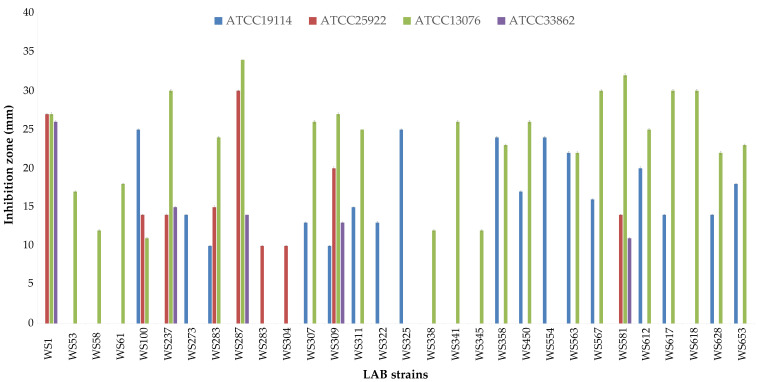
Inhibitory activity of LAB isolated from wooden shelf biofilms. Strain codes belong to the following species: *En. faecalis* (WS1), *En. faecium* WS237, WS273, WS283, WS311, WS322, WS325, WS338, WS341, WS345, WS358, WS554, WS563, WS612, and WS653) *En. durans* (WS304), *En. lactis* (WS288), *En. viikkiensis* (WS58), *Lc. lactis* (WS61), *Ln. mesenteroides* (WS53, WS307, WS567, WS581, WS618, and WS628), *P. pentosaceus* (WS287, WS309, and WS450), *Lb. delbrueckii* (WS100), *W. hellenica* (WS617), and *W. paramesenteroides* (WS581).

Among the dominant LAB, 75 strains were identified and allotted into the species *Enterococcus faecalis, Enterococcus faecium, Enterococcus durans, Enterococcus casseliflavus, Enterococcus gallinarum, Enterococcus italicus, Enterococcus lactis, Enterococcus thailandicus, Enterococcus viikkiensis, Lactococcus lactis, Leuconostoc mesenteroides, Pediococcus acidilactici, Pediococcus pentosaceus, Lactobacillus delbrueckii, Weissella hellenica*, and *Weissella paramesenteroides* [21]. In the present work, all LAB strains were tested for their inhibitory properties against the four main dairy pathogens. Thirty strains were able to inhibit at least one indicator strain (Figure 4).

In general, the most sensitive bacterium was *S.* Enteritidis ATCC13076, while the most resistant one *St. aureus* ATCC33862. Several enterococci were able to inhibit the undesired bacteria, but the highest inhibitory activity was registered for *P. pentosaceus* WS287 and *W. paramesenteroides* WS581 in terms of number of indicators inhibited and with of the clear areas.

## 4. Discussion

The use of wooden shelves in Sicily is mandatory for the ripening of several traditional semi-hard and hard cheeses, including PDO Pecorino Siciliano, PDO Piacentinu Ennese, and TAP Caciocavallo Palermitano [21]. These three cheeses are strongly linked to the production area. This implicates that the production environment exerts a defining role on the final cheese characteristics [2,3,4]. Although traditional cheeses produced in the Sicily region are not inoculated with starter cultures, the acidification and ripening processes rely on the indigenous LAB of raw materials or wooden vat biofilms [27,28,29]. This strongly indicates that the dairy environment of a given facility might influence the microbial evolution during cheese transformation. Furthermore, the microorganisms (especially bacteria) inside the cheese are characterized by a different spatial distribution due to the physicochemical characteristics of the different layers [30,31]. Although the complex network of interactions between biotic (microbial interactions) and abiotic (pH, water activity, redox potential, and chemical composition) factors within cheese that determine the microbial dynamics is poorly understood [32], the microorganisms located just under the rind have to be particularly resistant to high salt concentrations and low water activity. The rind of stretched and pressed cheeses is basically considered to be a barrier, playing no active role during cheese ripening [33]. However, Settanni et al. [21] supposed that the wooden boards used for the ripening of PDO Pecorino Siciliano, PDO Piacentinu Ennese, and TAP Caciocavallo Palermitano were able to transfer the bacteria responsible for the centripetal maturation. Thus, the present study was undertaken to evaluate the relationships among wooden shelf bacterial diversity and cheese typology, applying a multivariate statistical approach.

First of all, the microbial loads of the wooden shelf biofilms were subjected to a canonical discriminant analysis, which confirmed a cheese typology effect on the microbial loads of the 18 wooden shelves investigated. The three graphic areas identified in the plot of can 1 × can 2 were quite distant, especially thanks to the levels of the rod LAB. Basically, the longest statistical distances were measured between the wooden shelves used for ripening ovine cheeses (PDO Pecorino Siciliano and PDO Piacentinu Ennese) and those used for the bovine TAP Caciocavallo Palermitano cheese. Canonical correlation analysis is generally applied in the dairy environment to discriminate among sources of contamination of *Pseudomonas* [34], to investigate on the growth interaction between *St. aureus* and *Lactococcus lactis* subsp. *cremoris* in inoculated milk [35], and to correlate cheese volatile organic compounds with starter and non-starter LAB [36], indicating a wide field of application of this statistical approach in dairy science. Strictly regarding discrimination about cheeses, Manca et al. [37] demonstrated that a multivariate treatment of casein and amino acid data differentiated numerous samples of Pecorino Sardo, Pecorino Siciliano, and Pecorino Pugliese cheeses according to place of origin (Sardinia, Sicilia, and Apulia regions, respectively). Based on our results, a similar discrimination could be tempted exclusively with microbiological data, given that the biofilm levels could provide differentiation among cheese production. For this purpose, the levels of TMM found on the wooden boards used in Sicily for the three cheeses of the present investigation were lower than those detected on the shelves used to ripen Reblochon de Savoie cheese in France [17] or Fontina cheese in northern Italy [19]—both smear cheeses—but higher than those reported for the shelves used for ripening Serro and Canastra cheeses in Brazil [38]. These data confirm that the levels of biofilms on the wooden shelves were strictly related to the cheese typology.

The same statistical approach was used to correlate bacterial OTUs at the order level and for cheese typology. The most relevant results showed that the wooden shelves used for PDO Piacentinu Ennese cheese ripening were characterized by the highest presence of Flavobacteriales, while those used for TAP Caciocavallo Palermitano cheese ripening were characterized by the highest relative abundance of Bacillales. Generally, Flavobacteriales are identified as cheese biomarkers during raw milk cheddar cheese production [39]. Biomarkers are bacterial communities that are significantly and relatively highly abundant in two or more samples, that are useful to explain conditions of the sample source [40]. Bacillales are reported to dominate in raw milk during the warm seasons (spring and summer months) [41], and all of the TAP Caciocavallo Palermitano cheeses in contact with the wooden board analyzed in this study were produced during the summer season. Discriminant canonical analysis once again showed a good graphic separation among the three areas obtained from the data of the three cheeses analyzed, confirming the results obtained from the univariate analysis. The multivariate analysis of the metataxonomic and metafingerprinting data were successfully discriminated between dominant and subdominant taxa for the PDO Grana Padano cheese and generical hard cheeses [42], cheeses whose appearance is highly similar to that of the PDO Grana Padano cheese. Hence, our study represents a useful suggestion to also use the microbiota of the ripening tools to trace the typicality of PDO cheeses, and provides an additional measure to face their counterfeiting.

Generally, under the conditions of a given dairy facility, environmental contamination by LAB might be observed during cheese production [43]. So far, there is no evidence on what happens to the wooden shelf biofilms during cheese ripening; actually, knowledge about the microbial communities associated with cheese ripening wooden boards is limited [20]. In our opinion, there is a quite urgent need to fill this gap, especially after the so-called “cheese apocalypse” reported by Forbes in 2014, based on the FDA decree to answer the request from the New York State Department of Agriculture regarding the acceptability of wooden surfaces for cheese aging. The agency responded that “the use of wooden shelves, rough or otherwise, for cheese ripening does not conform to current good practices” [44], and issued an alert on the potential presence of pathogenic bacteria transferred by Italian and French cheeses ripened on wooden shelves [16]. In the present study, considering that the wooden shelves were sampled from several facilities characterized by their own environments, data from multivariate statistical elaboration highlighted how the microbial differences evaluated both in terms of viable levels and OTU classification among the board biofilms were mainly imputable to the cheese type, rather than the environmental factors of the facilities analyzed.

The absence of pathogenic species after culture-dependent and -independent microbiological investigations assumed an inhibiting role of the microbial populations of wooden board biofilms. A previous investigation demonstrated a certain potential of the wooden shelf LAB to inhibit *L. monocytogenes* [18]. Based on this consideration, all strains isolated and identified by Settanni et al. [21] from the wooden shelf biofilms characterized from PDO Pecorino Siciliano, PDO Piacentinu Ennese, and TAP Caciocavallo Palermitano cheeses were investigated for their ability to inhibit the growth of the main dairy pathogens, namely: *E. coli*, *L. monocytogenes*, *S.* *Enteritidis*, and *St. aureus*. The results clearly showed a definite activity against *S.* *Enteritidis* ATCC13076, while the most resistant strain was *St. aureus* ATCC33862. However, the most interesting result was that a huge number of wooden shelf LABs were able to inhibit at least one of the undesired bacteria. In general, it is known that LABs isolated from fermented foods, where adaptation to an environment rich in nutritional sources plays a major role for their persistence, are characterized by a very low ability to produce inhibitory substances, and that their raw materials host higher percentages of positive strains and a higher inhibitory activity [37,38]. This work provides further evidence to that of Mariani et al. [18] on the inhibitory spectrum of the LAB biofilms of wooden shelves used to ripen cheeses. Furthermore, it is worth noting that several enterococci were inhibitors of the indicator strains, but the highest activity was found for *P. pentosaceus* WS287 and *W. paramesenteroides* WS581. These results are particularly interesting because non-*Enterococcus* LAB have a wider application in dairy transformations than enterococci due to the innate presence of antibiotic resistance in several strains of the latter group [45].

## 5. Conclusions

The dual statistical approach (multivariate and univariate analysis) applied in this study clearly showed that the microbial levels and the bacterial composition of the biofilms of the wooden shelves used to ripen traditional cheeses are influenced by the cheese typology. This is relevant information retrieved from the levels of the 12 microbial groups analyzed and the bacterial classification based on OTUs’ attribution, in particular in consideration of the fact that all 18 facilities where wooden shelf biofilms were collected are characterized by quite unique environmental conditions. Furthermore, the biofilms associated with the wooden shelves investigated exerted an inhibitory activity against the main dairy pathogens, suggesting a barrier effect of LAB. In order to better and deeply investigate the development and composition of biofilms on the wooden shelves used to ripen traditional Sicilian cheeses, the effect of wood type, board age, and salting technology will be analyzed in future works. At present, works are being prepared to evaluate the in situ inhibitory activity of the selected strains showing the best performances in terms of indicator inhibition on virgin boards subjected to the pressure of artificially inoculated pathogenic strains.

## Figures and Tables

**Table 1:** Microbial loads (LSM ± s.e.) of the biofilms developed on the wooden shelves used to ripen traditional Sicilian cheeses.

Bacterial Counts	Cheeses			*p* Value
	TAP Caciocavallo Palermitano	PDO Piacentinu Ennese	PDO Pecorino Siciliano	
TMM	5.09 ± 0.26 b	4.97 ± 0.28 b	5.84 ± 0.24 a	<0.036
Enterobacteriaceae	0 ± 0 B	0.61 ± 0.27 B	1.79 ± 0.23 A	<0.001
Total coliforms	0 ± 0 B	0.46 ± 0.26 AB	1.08 ± 0.22 A	<0.005
*E. coli*	0 ± 0 Bb	0.34 ± 0.28 ABb	1.17 ± 0.24 Aa	<0.004
Pseudomonads	0.94 ± 0.40 B	3.96 ± 0.44 A	3.21 ± 0.37 A	<0.001
Enterococci	1.95 ± 0.17 B	3.14 ± 0.19 A	2.11 ± 0.16 B	<0.001
Mesophilic LAB rods	3.40 ± 0.31 B	3.32 ± 0.34 B	4.64 ± 0.29 A	<0.004
Thermophilic LAB rods	1.79 ± 0.28 A	3.09 ± 0.30 B	1.29 ± 0.26 A	<0.001
Mesophilic LAB cocci	4.78 ± 0.35	5.31 ± 0.39	5.60 ± 0.32	<0.234
Thermophilic LAB cocci	2.45 ± 0.30	3.19 ± 0.33	2.57 ± 0.28	<0.213
Yeasts	3.84 ± 0.28 Aa	5.07 ± 0.31 Bb	4.75 ± 0.26 ABb	<0.010
Molds	2.34 ± 0.35 AB	1.47 ± 0.39 B	3.12 ± 0.33 A	<0.007

Units are Log CFU/cm^2^. LSM—least-square method; s.e.—standard error; TMM—total mesophilic microorganisms; *E.*—*Escherichia*; LAB—lactic acid bacteria. In the rows, different capital letters are significant for *p* < 0.01; different letters are significant for *p* < 0.05.

**Table 2:** Standardized canonical discriminant function coefficients.

Variables	1st Canonical Variable	2nd Canonical Variable
TMM	−0.512	−1.141
Enterobacteriaceae	−1.843	0.503
Total coliforms	0.210	0.489
E. coli	0.388	−0.405
Pseudomonads	0.753	1.283
Enterococci	0.802	0.907
Mesophilic LAB rods	−2.042	0.192
Thermophilic LAB rods	1.390	−1.720
Mesophilic LAB cocci	1.091	−0.214
Thermophilic LAB cocci	−0.270	0.514
Yeasts	0.451	1.116
Molds	−0.282	0.017
Variance explained (%)	76.2	23.8

**Table 3:** Relative abundance (%) of operational taxonomy units (LSM ± s.e.).

Orders	Cheeses			*p* Value
	TAP Caciocavallo Palermitano	PDO Piacentinu Ennese	PDO Pecorino Siciliano	
Actinomycetales	26.85 ± 0.60	19.04 ± 0.82	34.78 ± 0.44	<0.609
Flavobacteriales	0.11 ± 0.46 B	6.84 ± 0.45 A	0.29 ± 0.46 B	<0.001
Sphingobacteriales	0.87 ± 0.47	0.06 ± 0.47	0.05 ± 0.48	<0.137
Bacillales	43.72 ± 0.09 a	16.39 ± 0.01 b	26.23 ± 0.15 ab	<0.050
Lactobacillales	6.10 ± 0.09	4.17 ± 0.22	9.05 ± 0.02	<0.698
Clostridiales	0.62 ± 0.47	0.56 ± 0.47	0.29 ± 0.82	<0.696
Rhizobiales	3.58 ± 0.22	0.00 ± 0.16	0.08 ± 0.26	<0.171
Alteromonadales	0.00 ± 0.38	2.85 ± 0.36	0.73 ± 0.40	<0.124
Enterobacteriales	0.02 ± 0.46	0.75 ± 0.45	0.27 ± 0.47	<0.420
Oceanospirillales	1.90 ± 0.04	10.46 ± 0.05	4.98 ± 0.10	<0.249
Pseudomonadales	0.08 ± 0.27	1.96 ± 0.22	1.69 ± 0.30	<0.453
Salinisphaerales	0.02 ± 0.21	2.67 ± 0.15	0.87 ± 0.25	<0.439
Rhodobacterales	0.08 ± 0.48	0.64 ± 0.47	0.08 ± 0.48	<0.296

LSM—least-square method; s.e.—standard error. In the rows, different capital letters are significant at *p* < 0.01; different letters are significant at *p* < 0.05.

**Table 4:** Standardized canonical discriminant function coefficients.

Variables	1st Canonical Variable	2nd Canonical Variable
Actinomycetales	3.459	1.071
Flavobacteriales	−6.860	0.759
Sphingobacteriales	−3.574	1.601
Bacillales	3.895	1.753
Lactobacillales	2.788	0.757
Clostridiales	1.818	−1.384
Rhizobiales	5.211	−0.016
Alteromonadales	−3.253	1.885
Enterobacteriales	−0.328	−2.622
Oceanospirillales	4.884	−0.349
Pseudomonadales	−0.275	−0.322
Salinisphaerales	−3.170	−0.082
Rhodobacterales	−0.142	3.242
Variance explained (%)	99.0	1.0

## Data Availability

All of the data included in this study are available upon request by contacting the corresponding author.
